# Subjective–objective fit in tennis racket string-tension selection: perceived comfort, hitting performance, and fastest swing-speed associations

**DOI:** 10.3389/fpsyg.2026.1889737

**Published:** 2026-08-13

**Authors:** Jun-jie Yang, Bo Sun, Jie Ren

**Affiliations:** Shanghai University of Sport, Shanghai, China

**Keywords:** ball speed, fastest racket swing speed, hitting accuracy, motor performance, perceived comfort, sport equipment, string tension, subjective–objective fit

## Abstract

**Background:**

Tennis racket string tension may influence ball speed, directional control, and perceived comfort, yet evidence from real hitting tasks remains limited.

**Objective:**

This study examined the effects of five string tensions on forehand and backhand performance and tested whether subjective comfort aligns with objectively optimal tension.

**Methods:**

Twenty-seven trained tennis players completed a repeated-measures experiment using five identical rackets strung at 40, 45, 50, 55, and 60 lb. Ball speed was derived from high-speed video, accuracy was scored using a concentric target, and perceived comfort was assessed using a 5-point Likert scale and final preferred-racket selection. Ball speed and accuracy were analyzed using 2 hitting sides × 5 string tensions × 2 sex groups mixed-design ANOVAs. Correlations examined associations among fastest racket swing speed, subjectively preferred tension, and individualized optimal-tension outcomes.

**Results:**

Forehand ball speed was significantly higher than backhand ball speed, *F*(1,25) = 41.51, *p* < 0.001, ηp^2^ = 0.624. String tension did not significantly affect ball speed, whereas accuracy differed significantly across tensions, *F*(4,100) = 40.37, *p* < 0.001, ηp^2^ = 0.618, generally improving at higher tensions. A significant tension × sex interaction was also observed for accuracy, *F*(4,100) = 4.22, *p* = 0.003, ηp^2^ = 0.144. Fastest racket swing speed was positively associated with ball speed at the speed-optimal tension for both strokes, but not with optimal-tension values or best accuracy scores. Subjectively preferred tension showed limited agreement with objective speed- or accuracy-optimal tension, and no correlations remained significant after FDR correction.

**Conclusion:**

Within 40–60 lb, string tension influenced accuracy more consistently than ball speed. Perceived comfort provides complementary information but should not replace objective performance testing in individualized racket configuration.

## 1 Introduction

Tennis is an interceptive racket sport in which players must rapidly integrate ball perception, anticipatory decision-making, footwork adjustment, racket acceleration, and impact control (Elliott, 2006; Hennig, 2007; Reid et al., 2013). Among tennis strokes, the forehand and backhand are fundamental actions for baseline rallies, attacking transitions, defensive recovery, and point construction. The forehand is often associated with greater offensive potential, whereas the backhand contributes substantially to rally stability, defensive pressure management, and bilateral stroke competence. Because forehand and backhand strokes differ in force-production structure, swing trajectory, body coordination, and racket-face control, the same equipment parameter may not influence the two strokes in identical ways. Examining forehand and backhand performance within the same experimental framework is therefore important for understanding how adjustable racket parameters affect tennis performance and equipment individualization.

String tension is one of the racket parameters most frequently manipulated by players, coaches, and stringers (Elliott, 1982; Bower and Sinclair, 1999; Brody et al., 2002; Cross, 2003; Cross and Lindsey, 2005). It directly affects string-bed stiffness, racket-face deformation, ball-racket contact time, and energy transfer during impact, which may in turn influence ball speed, outgoing trajectory, and directional control. In general, lower string tension is thought to increase string-bed deformation and ball dwell time, thereby facilitating elastic rebound and speed production, whereas higher string tension may create a stiffer string bed and clearer impact feedback, thereby supporting control. However, the relationship between string tension and performance is unlikely to follow a simple rule of “lower tension for speed and higher tension for control”; it may depend on racket structure, string material, stroke type, incoming-ball conditions, player skill level, and individual adaptation.

Previous studies have provided useful insights into the role of string tension in ball-racket interaction, but findings remain inconsistent across testing contexts (Elliott, 1982; Bower and Sinclair, 1999; Bower and Cross, 2003, 2005; Zhao et al., 2025). Laboratory impact studies indicate that reduced tension not only increases rebound speed but also alters specific collision characteristics, notably increasing the coefficient of restitution (COR), raising the rebound angle, and enhancing the ‘trampoline effect’ through greater string-bed deformation. In contrast, real or simulated hitting tasks introduce active player adaptation through swing speed, impact timing, and racket-face regulation (Bower and Cross, 2005; Zhao et al., 2025). Consequently, ball speed and landing error do not always change linearly with string tension. Some evidence also suggests that intermediate string tensions may provide a favorable balance between speed and control. These findings highlight the task-dependent nature of string-tension effects and indicate that real sport-specific hitting tasks are needed to complement laboratory impact evidence.

Several research gaps remain. First, many studies have emphasized forehand strokes, mechanical impact testing, or highly standardized mechanical simulations, whereas less is known about how trained players adapt both forehand and backhand performance to multiple tension conditions in real hitting tasks (Bower and Cross, 2005; Zhao et al., 2025). Second, previous studies often treat ball speed or accuracy as isolated outcomes. In practice, however, stroke quality is usually defined by a speed–accuracy trade-off: a faster ball is not necessarily a better shot, and greater control may not always coincide with maximal speed (Fitts, 1954; Schmidt et al., 1979). It is therefore necessary to examine ball speed and accuracy simultaneously when assessing the practical implications of varying string tensions.

Another issue closely related to sport psychology and movement science is that equipment selection is not based solely on objective performance (Bower and Cross, 2003; Stroede et al., 1999; Yeh et al., 2019; Ripberger et al., 2025; Piquer-Piquer et al., 2025). Athletes also rely on perceived comfort, impact feel, sense of control, and trust in the racket. These subjective and perceptual responses may influence athletes’ confidence in equipment, willingness to use a given setup consistently, and performance stability during training or competition (Farrow and Reid, 2010; Larson and Guggenheimer, 2013; Buszard et al., 2014, 2016; Piquer-Piquer et al., 2025). Perceived comfort should therefore be considered a meaningful equipment-user outcome. However, it remains unclear whether subjective preference corresponds to the objectively optimal tension for ball speed or accuracy. Examining the agreement and association between subjectively preferred tension and objectively optimal performance conditions may clarify whether perceived comfort can guide individualized racket configuration or is better interpreted as a complementary perceptual indicator (Ripberger et al., 2025; Piquer-Piquer et al., 2025). At the same time, individual hitting capacity may also affect adaptation to string tension.

Accordingly, under standardized racket, string, and ball-feeding conditions, this study compared forehand and backhand ball speed and hitting accuracy across five string tensions (40, 45, 50, 55, and 60 lb) in trained tennis players (Bower and Cross, 2005; Zhao et al., 2025). The study also examined whether forehand and backhand fastest racket swing speeds were associated with individualized optimal-tension outcomes, including the tension that produced the fastest ball speed, the ball speed obtained at that tension, the tension that produced the highest accuracy score, and the accuracy score obtained at that tension. Finally, the study analyzed whether subjectively preferred tension was consistent with, or associated with, objectively speed-optimal and accuracy-optimal tension outcomes. By integrating objective performance, individual swing capacity, and perceived comfort (Bower and Cross, 2003; Ripberger et al., 2025; Piquer-Piquer et al., 2025), this study aimed to provide empirical evidence for individualized string-tension selection and to strengthen the sport-psychological interpretation of equipment–user interaction in tennis.

Based on the mechanical and perceptual roles of string tension, we hypothesized that varying string tension would influence ball speed and hitting accuracy in different ways. Specifically, lower-to-intermediate tensions were expected to be associated with relatively higher ball speed because of greater string-bed deformation and elastic rebound, whereas medium-to-high tensions were expected to be associated with better hitting accuracy because of increased string-bed stiffness and clearer impact feedback. We further hypothesized that the tension associated with subjective comfort would not necessarily fully correspond to the objectively speed-optimal or accuracy-optimal tension, indicating a potential mismatch between subjective preference and objective performance.

## 2 Materials and methods

### 2.1 Participants

Twenty-seven trained tennis players from Shanghai University of Sport were recruited for this study. All participants had achieved at least the Chinese national second-level tennis athlete classification. The sample included 18 men and 9 women. Men were 23.6 ± 2.9 years old and had 9.1 ± 4.2 years of tennis training experience; women were 22.7 ± 2.7 years old and had 6.8 ± 3.4 years of tennis training experience. All participants had systematic tennis training experience, and 12 were first-level athletes. All participants were right-handed and used a two-handed backhand. None had experienced shoulder, elbow, wrist, or other sport injuries affecting test performance during the preceding 6 months. Before testing, all participants were informed of the study procedures, potential risks, and precautions, and provided written informed consent. The study was approved by the Scientific Research Ethics Committee of Shanghai University of Sport (approval No. 2025RT252) (Table 1).

**Table 1:** Participant characteristics.

Sex	n	Age (years)	Height (cm)	Body mass (kg)	Tennis training experience (years)
Men	18	23.6 ± 2.9	176.5 ± 5.7	72.89 ± 10.7	9.1 ± 4.2
Women	9	22.7 ± 2.7	167.2 ± 6.5	57.78 ± 6.7	6.8 ± 3.4

### 2.2 Study design

A repeated-measures experimental design was used. String tension was treated as a within-subject factor with five levels (40, 45, 50, 55, and 60 lb), and sex was included as a between-subject factor (Bower and Cross, 2005; Zhao et al., 2025). The dependent variables were forehand ball speed, backhand ball speed, forehand accuracy, and backhand accuracy. Forehand and backhand fastest racket swing speeds were also measured to characterize individual tennis-specific swing capacity. In addition to the repeated-measures performance analyses, individualized optimal-tension correlation analyses were conducted. These analyses were based on participant-level derived variables rather than on the original repeated-measures observations. Specifically, for each participant and each hitting side, the five tension-condition values were reduced to individualized optimal-tension indicators, including speed-optimal tension, ball speed at the speed-optimal tension, accuracy-optimal tension, and accuracy score at the accuracy-optimal tension. These derived variables were then used to examine whether individual swing capacity and subjectively preferred tension were associated with objectively optimal performance outcomes. Perceived comfort was assessed as a subjective outcome reflecting the athlete–equipment experience and was further used to examine subjective–objective fit with the derived optimal-tension outcomes.

### 2.3 Apparatus and experimental setting

Testing was conducted indoors under standardized conditions. Kinematic data were collected using two Vision Research (AMETEK) MIRQ-series high-speed cameras at a sampling frequency of 3,000 frames per second. These cameras recorded racket-head and ball motion during swing and hitting trials. Landing location was recorded using a high-definition digital camera positioned behind the target area.

Five Wilson Blade V8 rackets with identical specifications were used: 98 in^2^ (632 cm^2^) head size, 305 g unstrung mass, 16 × 19 string pattern, and grip size 2 (4 1/4 inches or 10.8 cm in circumference). All rackets were strung with TAAN 8800 polyester string (1.25 mm diameter). To ensure maximum tension precision and control, all rackets were strung using a professional electronic constant-pull stringing machine, which was strictly calibrated with a digital load cell immediately prior to the session. To mitigate and standardize the effects of early dynamic tension loss, all five rackets were strung in a single controlled session and tested within a standardized short time window, thereby ensuring that the nominal relative tension differentials (5-lb increments) were strictly preserved across all conditions. The only experimental difference among the rackets was string tension: 40, 45, 50, 55, and 60 lb (Brody et al., 2002; Cross and Lindsey, 2005; Zhao et al., 2025; Taraborrelli et al., 2019). Standard ODEAR Passion competition tennis balls were used, and an automatic ball feeder provided standardized incoming balls to minimize variability in feed location, spin, and speed. A NET PLAYZ tennis radar speed device was used only for immediate feedback to help participants maintain maximal hitting intention; ball-speed data used in the statistical analyses were derived from high-speed video.

The automatic ball feeder was configured as a controlled ball-release device rather than a propulsive launcher. The outlet height was fixed at 140 cm, and the linear distance from the outlet to the target curtain was maintained at 800 cm. For the forehand and backhand tasks, the feeder was placed diagonally in front of the participant’s hitting side; these two feeder positions were kept fixed throughout the experiment to ensure that the ball consistently fell into the predefined forehand and backhand hitting zones. Because participants differed in height, arm span, and habitual contact point, the physical location of the feeder was not adjusted for individual participants. Instead, during the familiarization period, each participant adjusted their own court position to identify and maintain a comfortable and natural individualized hitting point. Ball release was initiated by the participant on each trial according to their own rhythm, so the firing rate was self-paced rather than preset by the machine. After the participant-triggered command, the feeder executed a standardized 2-s delay and then released the ball vertically from the outlet. No horizontal exit velocity or machine-generated spin (e.g., topspin or backspin) was imposed; therefore, the incoming ball was determined by free fall and vertical rebound, minimizing variability in feed speed, spin, and direction.

The testing area was an indoor space measuring 11 m × 8 m. A 6 m × 4 m buffer curtain was placed at the far end for safety. A circular target board was placed at the center of the curtain for the accuracy task. The target board had a diameter of 1.30 m, and its bottom edge was positioned 0.914 m above the floor, corresponding to the height of a standard tennis net. This arrangement ensured that the effective scoring area was located above net height and represented net-clearing shots. The effective scoring area consisted of five concentric zones with radii of 10, 20, 30, 40, and 50 cm from the target center, scored from the center outward as 5, 4, 3, 2, and 1 points, respectively. The remaining outer margin of the target board was not used as a separate scoring zone. When the ball landed on a boundary between two zones, the higher score was assigned. Landing locations were verified through slow-motion video review. Only trials landing within the effective scoring area were analyzed.

### 2.4 Testing tasks

#### 2.4.1 Forehand and backhand fastest racket swing speed

Before formal testing, participants completed a standardized tennis-specific warm-up and performed 2–3 min of forehand and backhand practice swings to become familiar with the racket and testing environment. Fastest racket swing speed was defined as the maximum racket-head speed recorded during a maximal stroke-like swing without ball contact. A high-intensity micro-LED marker was securely attached to the top of the racket head at the 12 o’clock position on the outer frame. Two high-speed cameras were positioned beside the swing trajectory to capture racket-head movement. Participants then performed one maximal forehand swing and one maximal backhand swing. Video data were imported into SIMI Motion software, where racket-head marker trajectories were reconstructed and maximal racket-head speed was extracted for subsequent correlation analyses. Fastest racket swing speed was expressed in meters per second (m/s).

#### 2.4.2 Ball-speed test

The ball-speed test was conducted under the five string-tension conditions. The five rackets were coded non-sequentially from 1 to 5 (i.e., the codes did not correspond to the ascending or descending order of string tensions). To eliminate potential expectation or guessing bias, participants were explicitly informed prior to testing that the codes were arbitrarily assigned and carried no sequential or directional information regarding the tensions. Participants were aware only of the racket code, not the corresponding string tension. Each participant used a random draw to determine the order in which the five rackets were tested. During the test, the automatic ball feeder delivered standardized balls. With each racket, participants performed five maximal forehand shots and five maximal backhand shots. High-speed cameras recorded the ball trajectory immediately after impact, and ball speed was calculated from ball displacement and frame interval. Participants rested for 5 min after completing forehand and backhand trials with each racket before proceeding to the next racket.

For both forehand and backhand ball-speed trials, the same participant-triggered ball-release procedure, standardized 2-s delay, fixed feeder positions, and individualized standing-position adjustment described above were used. Participants initiated each feed only after they felt physically and psychologically ready for the next stroke.

#### 2.4.3 Accuracy test

The accuracy test was performed after the ball-speed test and followed the same randomized racket order and blinded racket coding. With each racket, participants performed five forehand and five backhand shots while attempting to hit the central target on the curtain. A digital camera recorded landing locations, and accuracy was scored according to the predefined concentric-zone system. To reduce fatigue and short-term movement fluctuations, a 5-min rest interval was provided between racket conditions. Grip style, stance, and individualized stroke mechanics were not rigidly standardized; instead, players were allowed to use their habitual technique while completing the same task demands, thereby increasing the ecological relevance of the results for tennis performance.

#### 2.4.4 Perceived comfort

Immediately after completing the forehand and backhand ball-speed tests under each tension condition, participants rated their perceived hitting comfort using a 5-point Likert scale. The question was: “How comfortable did this racket feel during hitting under this tension condition?” The response anchors were 1 = very uncomfortable, 2 = uncomfortable, 3 = neutral, 4 = comfortable, and 5 = very comfortable. The same question and response anchors were used for forehand and backhand ratings under each tension condition (Sullivan and Artino, 2013). The ball-speed trials were specifically selected for the comfort assessment because they required maximal physical effort, which maximizes ball-racket impact forces and frame vibrations, thereby providing the most pronounced tactile and mechanical feedback for judging equipment comfort. Furthermore, measuring comfort at this stage reduced potential cognitive bias during the subsequent accuracy test, where participants’ perceptual ratings of the racket might be influenced by their spatial success or failure in hitting the target rather than by the physical feel of the equipment. After completing all conditions, participants separately selected the racket they perceived as the most comfortable for forehand and backhand strokes. These preferred-racket choices were converted into subjectively preferred tensions according to the actual tension corresponding to each coded racket. Subjectively preferred tension was used to examine the subjective-objective fit between perceived comfort and objectively speed-optimal or accuracy-optimal tension outcomes.

### 2.5 Outcome measures and data processing

Fastest racket swing speed was defined as the maximal racket-head speed during a maximal forehand or backhand swing without ball contact. Ball speed was defined as the speed of the ball after impact during forehand or backhand shots under each tension condition. For each tension and hitting side, five valid maximal shots were recorded, and the maximum value was used as the speed outcome. Hitting accuracy was scored according to the target-zone system; higher scores indicated that the shot landed closer to the center. For each tension and hitting side, the mean score across five valid trials was used as the accuracy outcome. Perceived comfort scores reflected participants’ immediate subjective responses to each tension condition. For optimal-tension analyses, speed-optimal tension was defined as the string tension that produced the fastest ball speed for each participant and hitting side, whereas accuracy-optimal tension was defined as the string tension that produced the highest accuracy score. The ball speed and accuracy score corresponding to these optimal-tension conditions were also extracted. To avoid a systematic upward bias in optimal-tension coding, tied maximum values were handled using a neutral rule. When two or more string tensions produced the same maximum value, the average of the tied tension values was used as the participant-level optimal-tension value for descriptive statistics and correlation analyses. For frequency distributions, tied cases were reported as tied optimal-tension categories rather than being assigned to the highest tension. For agreement analyses, exact agreement was counted when the subjectively preferred tension matched any of the tied optimal-tension conditions; agreement within ±5 lb was counted when the preferred tension was within 5 lb of any tied optimal-tension condition. If a missed ball, clearly invalid shot, or unidentifiable video trajectory occurred, one additional trial was recorded to ensure five valid trials per condition.

### 2.6 Experimental procedure

**Figure 1:**
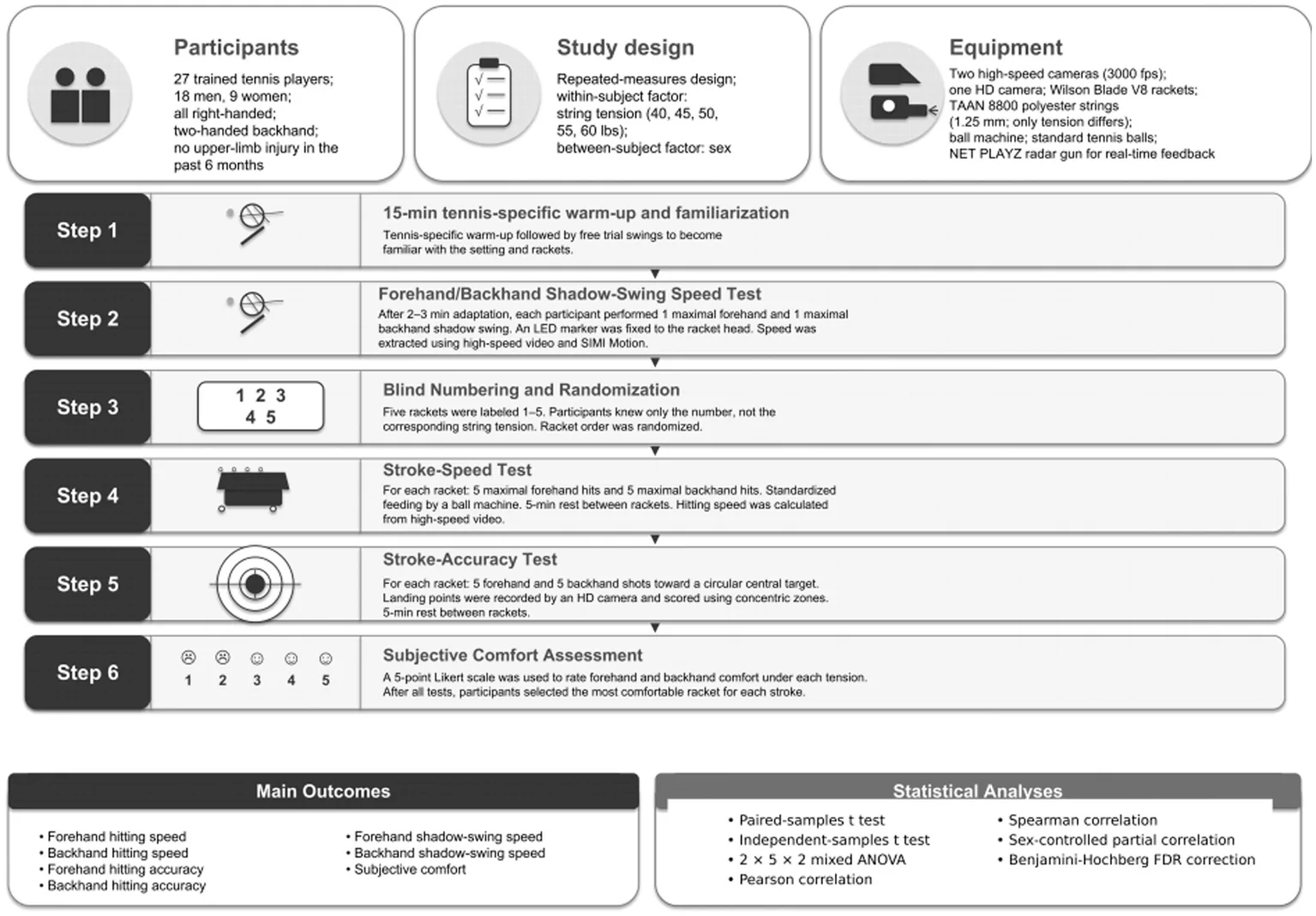
Schematic illustration of the experimental procedure for tennis racket performance testing.

To ensure testing consistency, all participants completed the tasks in the following order: warm-up and familiarization, swing-speed test, maximal ball-speed test, and accuracy test. All tests were performed in the same venue and with the same equipment. The order of the five string-tension rackets was randomized by draw. Participants first completed a 15-min tennis-specific warm-up and familiarization strokes within the testing area. A schematic illustration of the experimental procedure is presented in Figure 1.

### 2.7 Statistical analysis

Unless otherwise specified, data are presented as mean ± standard deviation. Statistical analyses were conducted using SPSS. Paired-samples t tests were used to compare forehand and backhand fastest racket swing speeds, and independent-samples t tests were used to examine sex differences in fastest racket swing speed. Ball speed and accuracy were analyzed using 2 hitting sides (forehand and backhand) × 5 string tensions (40, 45, 50, 55, and 60 lb) × 2 sex groups (male and female) mixed-design analyses of variance. Hitting side and string tension were treated as within-subject factors, whereas sex was treated as a between-subject factor. These models were used to examine whether string tension affected hitting performance and whether tension-response patterns differed by hitting side or sex.

For effects involving three or more within-subject levels, Mauchly’s test was used to assess sphericity. When the assumption of sphericity was violated, Greenhouse–Geisser-adjusted degrees of freedom and *p* values were reported (Greenhouse and Geisser, 1959). When a significant omnibus main effect or interaction was observed, follow-up simple-effects analyses and pairwise comparisons with Bonferroni adjustment were conducted where appropriate. Significant interactions involving sex and string tension were further visualized using interaction plots to aid interpretation.

For individualized optimal-tension analyses, participant-level derived variables were extracted separately for forehand and backhand strokes. Speed-optimal tension was defined as the string tension that produced the highest ball speed for a given participant and hitting side, and accuracy-optimal tension was defined as the string tension that produced the highest accuracy score. All individualized optimal-tension variables used in descriptive statistics, agreement analyses, and correlation analyses were calculated using the neutral tie-handling rule described above. Specifically, when two or more string tensions produced the same maximum value, the average of the tied tension values was used for descriptive statistics and correlation analyses. For frequency distributions, tied cases were retained as tied categories. For agreement analyses, exact agreement was counted when the subjectively preferred tension matched any of the tied optimal-tension conditions, and agreement within ±5 lb was counted when the preferred tension was within 5 lb of any tied optimal-tension condition. This approach avoided artificially shifting the optimal-tension variables toward the upper end of the tested tension range.

To examine the relationship between individual swing capacity and optimal-tension outcomes, forehand and backhand fastest racket swing speeds were separately correlated with four corresponding variables: speed-optimal tension, ball speed at the speed-optimal tension, accuracy-optimal tension, and accuracy score at the accuracy-optimal tension. Because optimal-tension variables were discrete and ties could occur, Spearman rank correlations were used as the primary analyses (Akoglu, 2018). Pearson correlations and sex-controlled partial correlations were conducted for continuous outcomes as sensitivity analyses.

To analyze subjective–objective fit between perceived comfort and objective performance, each participant’s final most-comfortable racket choice was converted into subjectively preferred tension and compared with objectively speed-optimal and accuracy-optimal tension outcomes using agreement and correlation analyses. Agreement was described using exact agreement, agreement within ±5 lb, and the direction of disagreement. Spearman correlations were used for the primary association analyses, and partial Spearman sensitivity analyses were conducted after rank transformation while controlling for sex. The Benjamini–Hochberg false discovery rate procedure was applied to each family of primary correlation tests to control for multiple testing (Benjamini and Hochberg, 1995). Statistical significance was set at a two-sided *α* level of 0.05. Exact *p* values are reported to three decimal places, and values smaller than 0.001 are reported as *p* < 0.001. Effect sizes were reported where appropriate (Lakens, 2013).

## 3 Results

### 3.1 Preliminary analyses and descriptive hitting performance

Forehand fastest racket swing speed was significantly higher than backhand fastest racket swing speed (34.85 ± 4.06 vs. 31.71 ± 3.37 m/s), with a mean difference of 3.15 m/s, 95% CI [2.10, 4.19], t(26) = 6.189, *p* < 0.001, Cohen’s d = 1.19. Fastest racket swing speed also showed significant sex differences. Men had higher forehand fastest racket swing speed than women (37.12 ± 2.07 vs. 30.30 ± 3.08 m/s), t(25) = 6.842, *p* < 0.001, Cohen’s d = 2.79; men also had higher backhand fastest racket swing speed than women (33.04 ± 3.12 vs. 29.04 ± 2.05 m/s), t(25) = 3.467, *p* = 0.002, Cohen’s d = 1.41. These findings support the inclusion or control of sex in subsequent analyses.

As shown in Table 2, forehand ball speed was highest under the 50-lb condition (147.8 ± 19.6 km/h) and lowest under the 60-lb condition (143.0 ± 19.1 km/h). Backhand ball speed was highest under the 45-lb condition (130.9 ± 12.4 km/h) and lowest under the 60-lb condition (125.7 ± 11.5 km/h). For accuracy, forehand scores increased progressively from 3.1 ± 0.6 points at 40 lb to 3.9 ± 0.4 points at 60 lb. Backhand accuracy increased from 3.0 ± 0.5 points at 40 lb to 3.7 ± 0.5 points at 55 lb and remained relatively high at 60 lb (3.6 ± 0.5 points).

**Table 2:** Descriptive statistics for ball speed and accuracy under each string-tension condition.

String tension	Forehand ball speed (km/h)	Backhand ball speed (km/h)	Forehand accuracy (points)	Backhand accuracy (points)
40 lb	143.7 ± 19.1	126.5 ± 11.7	3.1 ± 0.6	3.0 ± 0.5
45 lb	145.3 ± 19.1	130.9 ± 12.4	3.4 ± 0.6	3.2 ± 0.5
50 lb	147.8 ± 19.6	129.6 ± 11.7	3.5 ± 0.6	3.5 ± 0.4
55 lb	144.6 ± 19.6	127.0 ± 11.2	3.8 ± 0.5	3.7 ± 0.5
60 lb	143.0 ± 19.1	125.7 ± 11.5	3.9 ± 0.4	3.6 ± 0.5

Values are mean ± standard deviation. Ball speed was defined as the maximum value from five valid trials under each condition. Accuracy was defined as the mean score from five valid trials under each condition.

### 3.2 Effects of string tension on ball speed and accuracy

Table 3 shows that, for ball speed, the main effect of hitting side was significant, *F*(1,25) = 41.51, *p* < 0.001, ηp^2^ = 0.624, indicating that forehand ball speed was overall higher than backhand ball speed. The main effect of string tension was not significant, *F*(2.56,64.07) = 1.35, *p* = 0.269, ηp^2^ = 0.051; the hitting side × tension interaction was also not significant, *F*(1.98,49.44) = 1.96, *p* = 0.152, ηp^2^ = 0.073, indicating that the statistical pattern of ball-speed change across tensions did not differ significantly between forehand and backhand strokes. The hitting side × tension × sex interaction was not significant, F(1.98,49.44) = 0.53, *p* = 0.589, ηp^2^ = 0.021; however, the hitting side × sex interaction was significant, *F*(1,25) = 12.24, *p* = 0.002, ηp^2^ = 0.329, suggesting that the magnitude of the forehand–backhand speed difference differed by sex. Condition-specific *post hoc* comparisons showed that forehand ball speed was highest at 50 lb and was significantly higher than at 40 lb and 60 lb; backhand ball speed was highest at 45 lb and was significantly higher than at 40 lb and 60 lb. Because the overall hitting side × tension interaction was not significant, these post hoc comparisons should be interpreted as condition-specific differences rather than evidence of different optimal tension patterns for forehand and backhand strokes.

**Table 3:** Results of the mixed-design ANOVAs for ball speed and accuracy.

Outcome	Effect	F(df1, df2)	*p*	ηp^2^
Ball speed	Hitting side	41.51 (1, 25)	< 0.001	0.624
Ball speed	String tension	1.35 (2.56, 64.07)	0.269	0.051
Ball speed	Hitting side × tension	1.96 (1.98, 49.44)	0.152	0.073
Ball speed	Hitting side × tension × sex	0.53 (1.98, 49.44)	0.589	0.021
Ball speed	Hitting side × sex	12.24 (1, 25)	0.002	0.329
Accuracy	Hitting side	3.50 (1, 25)	0.073	0.123
Accuracy	String tension	40.37 (4, 100)	< 0.001	0.618
Accuracy	Tension × sex	4.22 (4, 100)	0.003	0.144
Accuracy	Hitting side × tension	0.72 (2.76, 68.88)	0.533	0.028
Accuracy	Hitting side × tension × sex	2.25 (2.76, 68.88)	0.095	0.083
Accuracy	Sex	0.01 (1, 25)	0.942	< 0.001

**Figure 2:**
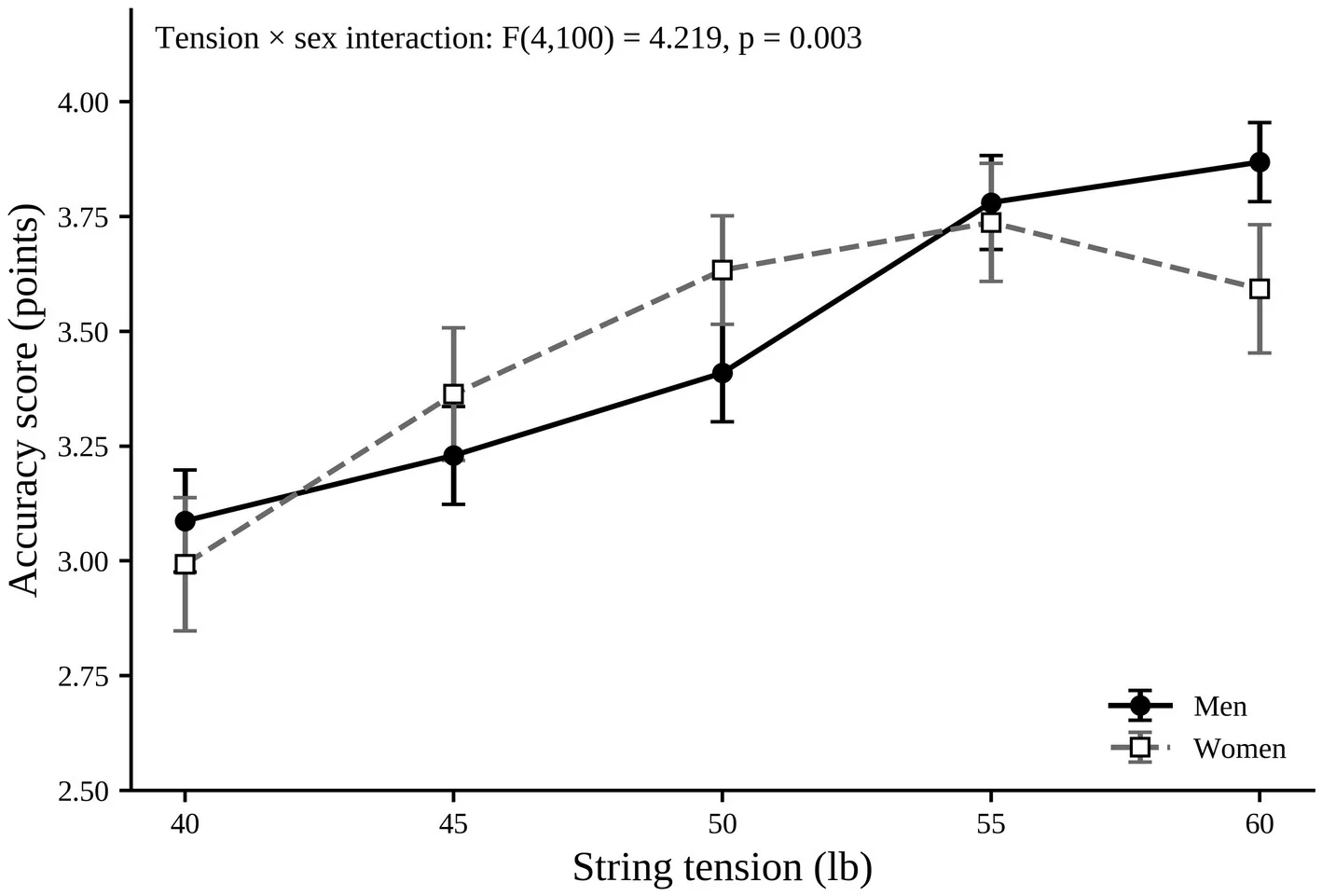
Interaction plot of accuracy scores by sex across the five string-tension levels. Accuracy scores were averaged across forehand and backhand conditions; error bars represent SEM.

For accuracy, Table 3 shows a significant main effect of string tension, *F*(4,100) = 40.37, p < 0.001, ηp^2^ = 0.618, indicating that accuracy differed significantly across the five tension conditions. In conjunction with Table 2, accuracy generally improved as tension increased. The tension × sex interaction was significant, F(4,100) = 4.22, *p* = 0.003, ηp^2^ = 0.144, suggesting different accuracy-response patterns in men and women. In contrast, the main effect of hitting side did not reach significance, F(1,25) = 3.50, *p* = 0.073, ηp^2^ = 0.123; the hitting side × tension interaction was not significant, *F*(2.76,68.88) = 0.72, *p* = 0.533, ηp^2^ = 0.028; and the hitting side × tension × sex interaction also did not reach significance, F(2.76,68.88) = 2.25, *p* = 0.095, ηp^2^ = 0.083. Simple-effects analyses were used to describe the significant tension × sex interaction, and Figure 2 visualizes the interaction after averaging forehand and backhand accuracy scores within each tension condition.

### 3.3 Associations between fastest racket swing speed and optimal-tension outcomes

For each participant, the tension corresponding to the fastest ball speed and the tension corresponding to the highest accuracy score were extracted separately for forehand and backhand strokes. When tied maximum values occurred, individualized optimal-tension variables were calculated using the neutral tie-handling rule described in the Methods section. Table 4 shows that the mean forehand speed-optimal tension was 50.4 ± 3.4 lb and was concentrated mainly at 50 lb (15 participants, 55.6%); ball speed at the forehand speed-optimal tension was 149.3 ± 19.4 km/h. Using the neutral tie-handling rule, the mean forehand accuracy-optimal tension was 57.3 ± 2.9 lb; the frequency distribution included 60 lb (13 participants, 48.1%), 55 lb (11 participants, 40.7%), 50 lb (1 participant, 3.7%), a 50/60-lb tied category (1 participant, 3.7%), and a 55/60-lb tied category (1 participant, 3.7%). The corresponding best accuracy score was 4.1 ± 0.4 points. The mean backhand speed-optimal tension was 47.0 ± 2.9 lb and was concentrated mainly at 45 lb (17 participants, 63.0%) and 50 lb (9 participants, 33.3%); ball speed at the backhand speed-optimal tension was 132.7 ± 11.6 km/h. Using the neutral tie-handling rule, the mean backhand accuracy-optimal tension was 55.6 ± 3.1 lb; the frequency distribution included 55 lb (11 participants, 40.7%), a 50/55-lb tied category (7 participants, 25.9%), 60 lb (7 participants, 25.9%), 50 lb (1 participant, 3.7%), and a 55/60-lb tied category (1 participant, 3.7%). The corresponding best accuracy score was 3.9 ± 0.4 points.

**Table 4:** Descriptive statistics for individualized optimal-tension variables.

Hitting side	Variable	Mean ± SD	Median (IQR)	Range	Frequency distribution of tension variables
Forehand	Fastest racket swing speed (m/s)	34.9 ± 4.1	36.4 (32.4–38.1)	25.2–39.6	—
Forehand	Speed-optimal tension (lb)	50.4 ± 3.4	50.0 (50.0–52.5)	45.0–55.0	45 lb: 5 (18.5%); 50 lb: 15 (55.6%); 55 lb: 7 (25.9%)
Forehand	Ball speed at speed-optimal tension (km/h)	149.3 ± 19.4	152.6 (137.4–163.5)	118.2–188.0	—
Forehand	Accuracy-optimal tension (lb)	57.3 ± 2.9	57.5 (55.0–60.0)	50.0–60.0	50 lb: 1 (3.7%); 55 lb: 11 (40.7%); 50/60 lb tie: 1 (3.7%); 55/60 lb tie: 1 (3.7%); 60 lb: 13 (48.1%)
Forehand	Accuracy score at accuracy-optimal tension (points)	4.1 ± 0.4	4.2 (3.9–4.3)	3.2–4.8	—
Backhand	Fastest racket swing speed (m/s)	31.7 ± 3.4	31.9 (29.2–34.4)	24.6–37.4	—
Backhand	Speed-optimal tension (lb)	47.0 ± 2.9	45.0 (45.0–50.0)	45.0–55.0	45 lb: 17 (63.0%); 50 lb: 9 (33.3%); 55 lb: 1 (3.7%)
Backhand	Ball speed at speed-optimal tension (km/h)	132.7 ± 11.6	131.6 (123.6–140.9)	112.0–151.0	—
Backhand	Accuracy-optimal tension (lb)	55.6 ± 3.1	55.0 (52.5–58.8)	50.0–60.0	50 lb: 1 (3.7%); 50/55 lb tie: 7 (25.9%); 55 lb: 11 (40.7%); 55/60 lb tie: 1 (3.7%); 60 lb: 7 (25.9%)
Backhand	Accuracy score at accuracy-optimal tension (points)	3.9 ± 0.4	4.0 (3.6–4.2)	3.0–4.6	—

Tension variables are expressed in pounds (lb). The em dash indicates not applicable. When tied optimal tensions occurred, the average of the tied tension values was used for mean, median, range, and correlation analyses; tied cases were retained as tied categories in the frequency distribution.

As shown in Table 5, Spearman correlations indicated that forehand fastest racket swing speed was significantly and positively associated with ball speed at the forehand speed-optimal tension, *ρ* = 0.718, 95% CI [0.465, 0.863], *p* < 0.001. Backhand fastest racket swing speed was also significantly and positively associated with ball speed at the backhand speed-optimal tension, ρ = 0.692, 95% CI [0.423, 0.849], p < 0.001. In contrast, forehand fastest racket swing speed was not significantly associated with speed-optimal tension (ρ = 0.123, *p* = 0.541), accuracy-optimal tension after neutral tie handling (ρ = 0.178, *p* = 0.374), or the accuracy score at the accuracy-optimal tension (ρ = 0.167, *p* = 0.404). Backhand fastest racket swing speed also did not reach significance for speed-optimal tension (ρ = −0.303, *p* = 0.124), accuracy-optimal tension after neutral tie handling (ρ = 0.276, *p* = 0.164), or the accuracy score at the accuracy-optimal tension (ρ = 0.323, *p* = 0.100).

**Table 5:** Spearman correlations between fastest racket swing speed and optimal-tension outcomes.

Hitting side	Predictor	Outcome	*n*	ρ	95% CI	*p*
Forehand	Fastest racket swing speed (m/s)	Speed-optimal tension (lb)	27	0.123	[−0.270, 0.481]	0.541
Forehand	Fastest racket swing speed (m/s)	Ball speed at speed-optimal tension (km/h)	27	0.718	[0.465, 0.863]	< 0.001
Forehand	Fastest racket swing speed (m/s)	Accuracy-optimal tension (lb)	27	0.178	[−0.239, 0.571]	0.374
Forehand	Fastest racket swing speed (m/s)	Accuracy score at accuracy-optimal tension (points)	27	0.167	[−0.227, 0.515]	0.404
Backhand	Fastest racket swing speed (m/s)	Speed-optimal tension (lb)	27	−0.303	[−0.613, 0.087]	0.124
Backhand	Fastest racket swing speed (m/s)	Ball speed at speed-optimal tension (km/h)	27	0.692	[0.423, 0.849]	< 0.001
Backhand	Fastest racket swing speed (m/s)	Accuracy-optimal tension (lb)	27	0.276	[−0.106, 0.593]	0.164
Backhand	Fastest racket swing speed (m/s)	Accuracy score at accuracy-optimal tension (points)	27	0.323	[−0.065, 0.626]	0.100

The sensitivity analyses shown in Table 6 were generally consistent with the primary analyses. Pearson correlations showed that forehand fastest racket swing speed was significantly associated with ball speed at the forehand speed-optimal tension, r = 0.769, *p* < 0.001, FDR q < 0.001; backhand fastest racket swing speed was also significantly associated with ball speed at the backhand speed-optimal tension, r = 0.656, *p* < 0.001, FDR q < 0.001. For the accuracy score at the accuracy-optimal tension, neither forehand (r = 0.176, *p* = 0.379, FDR q = 0.379) nor backhand (r = 0.314, *p* = 0.111, FDR q = 0.148) showed a significant association. After controlling for sex, the partial correlation between forehand fastest racket swing speed and ball speed at the speed-optimal tension was no longer significant, r = 0.328, *p* = 0.095. For the backhand, the corresponding partial correlation was significant before FDR correction, r = 0.425, *p* = 0.027, but should not be interpreted as robustly significant after multiple-comparison correction. These results indicate that the relationship between fastest racket swing speed and speed-output performance was partly influenced by sex-related differences.

**Table 6:** Sensitivity analysis for continuous outcomes.

Hitting side	Continuous outcome	Pearson r	*p*	FDR q	Sex-controlled partial r	*p*
Forehand	Ball speed at speed-optimal tension (km/h)	0.769	< 0.001	< 0.001	0.328	0.095
Forehand	Accuracy score at accuracy-optimal tension (points)	0.176	0.379	0.379	−0.024	0.904
Backhand	Ball speed at speed-optimal tension (km/h)	0.656	< 0.001	< 0.001	0.425	0.027
Backhand	Accuracy score at accuracy-optimal tension (points)	0.314	0.111	0.148	0.243	0.221

### 3.4 Subjectively preferred tension and objectively optimal-tension outcomes

Subjectively preferred tension was derived from each participant’s final choice of the most comfortable racket and was compared with objectively speed-optimal and accuracy-optimal tension outcomes. Descriptively, the mean subjectively preferred tension was 51.3 ± 7.7 lb for the forehand and 51.1 ± 8.1 lb for the backhand. Forehand subjective preference was concentrated at 55 lb and 60 lb, with 15 participants (55.6%) selecting one of these higher-tension conditions; for the backhand, 60 lb was selected most frequently, by 9 participants (33.3%).

Table 7 further shows limited agreement between subjectively preferred tension and objectively optimal tension conditions. Exact agreement with speed-optimal tension was 29.6% (8 participants) for the forehand and 14.8% (4 participants) for the backhand, whereas agreement within ±5 lb was 48.1% (13 participants) for both sides. Regarding the direction of disagreement, 8 participants (29.6%) showed subjectively lower tension and 11 participants (40.7%) showed subjectively higher tension than the forehand speed-optimal tension; the corresponding numbers for the backhand were 9 participants (33.3%) and 14 participants (51.9%). Compared with accuracy-optimal tension, exact agreement was slightly higher but still limited: 40.7% (11 participants) for the forehand and 25.9% (7 participants) for the backhand, with agreement within ±5 lb of 59.3% (16 participants) for both sides. For accuracy-optimal tension, a relatively high proportion of participants preferred tensions lower than their objectively optimal accuracy tension: 51.9% (14 participants) for the forehand and 55.6% (15 participants) for the backhand. This suggests that some players perceived a tension lower than their accuracy-optimal tension as most comfortable.

**Table 7:** Agreement between subjectively preferred tension and objectively optimal tension conditions.

Hitting side	Objective comparison	Exact agreement *n* (%)	Agreement within ±5 lb *n* (%)	Subjective lower *n* (%)	Subjective higher *n* (%)
Forehand	Speed-optimal tension	8 (29.6%)	13 (48.1%)	8 (29.6%)	11 (40.7%)
Forehand	Accuracy-optimal tension	11 (40.7%)	16 (59.3%)	14 (51.9%)	2 (7.4%)
Backhand	Speed-optimal tension	4 (14.8%)	13 (48.1%)	9 (33.3%)	14 (51.9%)
Backhand	Accuracy-optimal tension	7 (25.9%)	16 (59.3%)	15 (55.6%)	5 (18.5%)

Exact agreement indicates that subjectively preferred tension and objectively optimal tension were identical. Agreement within ±5 lb indicates that the absolute difference was no greater than 5 lb. Subjective lower/higher categories indicate the direction of disagreement relative to the objectively optimal tension.

As shown in Table 8, subjectively preferred tension was not significantly correlated with any objectively optimal-tension outcome after FDR correction. For the forehand, Spearman correlations between subjectively preferred tension and speed-optimal tension, ball speed at the speed-optimal tension, accuracy-optimal tension, and accuracy score at the accuracy-optimal tension were *ρ* = 0.037 (*p* = 0.853, FDR q = 0.853), ρ = 0.357 (*p* = 0.068, FDR q = 0.282), ρ = 0.041 (*p* = 0.840, FDR q = 0.853), and ρ = 0.346 (*p* = 0.077, FDR q = 0.282), respectively. For the backhand, the corresponding correlations were ρ = −0.291 (*p* = 0.141, FDR q = 0.282), ρ = 0.169 (*p* = 0.400, FDR q = 0.640), ρ = 0.315 (*p* = 0.110, FDR q = 0.282), and ρ = 0.096 (*p* = 0.635, FDR q = 0.846), respectively. Sex-controlled partial Spearman analyses led to the same conclusion, with no FDR-corrected *p* value reaching significance. Therefore, the tension perceived as most comfortable should be interpreted as a subjective equipment-preference indicator rather than a direct substitute for objective speed or accuracy testing.

**Table 8:** Associations between subjectively preferred tension and objectively optimal-tension outcomes.

Hitting side	Objective outcome	n	Spearman ρ [95% CI]	p (FDR q)	Sex-controlled partial ρ	p (FDR q)
Forehand	Speed-optimal tension (lb)	27	0.037 [−0.308, 0.383]	0.853 (0.853)	0.025	0.901 (0.959)
Forehand	Ball speed at speed-optimal tension (km/h)	27	0.357 [0.021, 0.627]	0.068 (0.282)	0.344	0.079 (0.432)
Forehand	Accuracy-optimal tension (lb)	27	0.041 [−0.380, 0.427]	0.840 (0.853)	−0.028	0.889 (0.959)
Forehand	Accuracy score at accuracy-optimal tension	27	0.346 [0.005, 0.638]	0.077 (0.282)	0.316	0.108 (0.432)
Backhand	Speed-optimal tension (lb)	27	−0.291 [−0.637, 0.081]	0.141 (0.282)	−0.242	0.223 (0.446)
Backhand	Ball speed at speed-optimal tension (km/h)	27	0.169 [−0.194, 0.498]	0.400 (0.640)	0.010	0.959 (0.959)
Backhand	Accuracy-optimal tension (lb)	27	0.315 [−0.047, 0.617]	0.110 (0.282)	0.261	0.188 (0.446)
Backhand	Accuracy score at accuracy-optimal tension	27	0.096 [−0.296, 0.457]	0.635 (0.846)	0.051	0.800 (0.959)

The primary analysis used Spearman rank correlations. The 95% CIs were estimated using bootstrap resampling. Partial correlations were estimated after rank transformation while controlling for sex. FDR q values were calculated using the Benjamini–Hochberg procedure across eight tests.

## 4 Discussion

This study examined forehand and backhand ball speed, hitting accuracy, individualized optimal-tension outcomes, and the subjective-objective fit between perceived comfort and objective performance under five tennis racket string-tension conditions in trained players. Five main interpretations emerged. First, forehand ball speed was higher than backhand ball speed overall, but string tension did not produce a significant overall effect on ball speed and did not show a significant hitting side × tension interaction. Second, accuracy was more consistently influenced by string tension, with higher tensions generally associated with better landing accuracy in the present task. Third, the significant tension × sex interaction for accuracy should be interpreted as a preliminary and exploratory pattern because the sample was relatively small and the sex distribution was unbalanced. Fourth, when a neutral tie-handling rule was used for individualized optimal-tension coding, fastest racket swing speed was associated with the ball speed achieved at the speed-optimal tension but not with the numerical value of the optimal tension or with accuracy-related optimal-tension outcomes. Fifth, perceived comfort and subjectively preferred tension showed limited agreement with objectively optimal-tension outcomes, indicating that perceived comfort is meaningful for equipment-user experience but should not be treated as a direct substitute for objective performance testing.

### 4.1 String tension and ball speed

Forehand ball speed was significantly higher than backhand ball speed, which is consistent with the movement characteristics of tennis strokes. Compared with the backhand, the forehand often allows greater trunk rotation, a larger swing path, and a more favorable upper-limb force-production structure, all of which may contribute to higher racket-head speed and ball speed. The finding that forehand fastest racket swing speed exceeded backhand fastest racket swing speed supports this interpretation from the perspective of side-specific movement capacity (Elliott, 2006; Reid et al., 2013).

In contrast, the mixed-design ANOVA did not show a significant main effect of string tension on ball speed, nor was there a significant hitting side × tension interaction. Thus, within the 40–60 lb range tested in this study, changing string tension did not produce a stable overall difference in ball speed, and the statistical pattern of ball-speed change across tensions did not differ significantly between forehand and backhand strokes. Condition-specific *post hoc* differences should therefore be interpreted cautiously as descriptive or condition-specific patterns rather than as evidence of distinct forehand and backhand optimal-tension profiles.

A plausible explanation is that ball speed in real hitting tasks reflects both racket-string mechanics and active player regulation. Mechanically, lower tension may increase string-bed deformation and elastic rebound, whereas higher tension may increase string-bed stiffness and shorten ball-racket contact time. However, players are not passive recipients of ball-racket collision mechanics; they regulate swing speed, impact timing, body positioning, and racket-face orientation during each stroke. The standardized ball-release procedure used here reduced incoming-ball variability, but it also created a controlled hitting context in which participants could adapt their position and stroke execution. Such adaptive regulation may reduce or mask the direct effect of string tension on ball speed during sport-specific hitting tasks (Elliott, 1982; Bower and Sinclair, 1999; Brody et al., 2002; Cross, 2003; Bower and Cross, 2003, 2005; Cross and Lindsey, 2005; Zhao et al., 2025).

### 4.2 String tension and accuracy

Compared with ball speed, accuracy showed a clearer response to string tension. Accuracy scores were lower under lower-tension conditions and generally improved under medium-to-high tension conditions. This pattern supports the hypothesis that higher string-bed stiffness may contribute more directly to landing control than to speed production in trained players. Because the hitting side × tension interaction for accuracy was not significant, the effect of string tension on accuracy should be interpreted as a general pattern across forehand and backhand strokes rather than as evidence that the two hitting sides responded differently to changes in string tension.

One possible explanation is that higher string tension increases string-bed stiffness and alters the sensory feedback associated with impact. Through prior experience and repeated exposure, players may learn to use this feedback to adjust pre-impact racket-face orientation, timing, and stroke execution on subsequent strokes, rather than regulating the outgoing ball during the brief ball–string contact period. Compared with ball speed, accuracy depends more strongly on racket-face angle, impact stability, timing, and perceptual feedback at contact. Therefore, accuracy may be more sensitive to string-bed stiffness and perceived impact clarity. The present pattern, in which ball speed was not consistently affected by tension whereas accuracy was significantly affected, suggests that string tension may influence control-related performance more reliably than speed-related performance under the present testing conditions (Brody et al., 2002; Cross and Lindsey, 2005; Bower and Cross, 2005; Ripberger et al., 2025; Zhao et al., 2025).

The descriptive pattern also suggests that accuracy benefits may become more evident from approximately 50 lb upward, particularly for backhand accuracy, which remained relatively high at 55 lb and 60 lb. This interpretation is discussed here rather than treated as a confirmatory result because descriptive plateau patterns should not be overemphasized beyond the omnibus model and follow-up analyses. Future studies with larger samples and trial-level models should test whether such apparent plateaus represent a stable control benefit or merely sample-specific variation.

### 4.3 Preliminary sex-related accuracy patterns and practical interpretation

The significant tension × sex interaction for accuracy should be interpreted cautiously because the sample was relatively small and the sex distribution was unbalanced, with 18 men and 9 women. Accordingly, the sex-related patterns observed in this study should be regarded as preliminary and hypothesis-generating rather than as definitive evidence of sex-specific adaptation. The interaction plot helps visualize the observed pattern, but it should be considered an aid to interpretation rather than independent confirmation of a stable sex-specific effect.

In men, forehand accuracy appeared higher at 55 lb and 60 lb than at lower tensions, suggesting a possible plateau around 55 lb. In women, forehand accuracy appeared to improve from 40 lb and to plateau from approximately 50 lb onward. These patterns suggest that the tension range associated with higher accuracy may differ between men and women in this sample, but this interpretation requires confirmation in larger and more balanced samples. Therefore, the present findings should not be used as a definitive basis for sex-specific string-tension prescriptions.

From an applied perspective, these preliminary observations still have practical value because they highlight the need for individualized racket fitting rather than reliance on a single universal tension. Sex-related patterns may be considered cautiously, but individual swing capacity, stroke habits, perceived comfort, and actual speed-accuracy performance should remain central to equipment prescription (Hennig, 2007; Bower and Cross, 2005; Zhao et al., 2025; Piquer-Piquer et al., 2025).

### 4.4 Individual swing capacity and optimal-tension outcomes

Analyses based on individualized optimal-tension indicators examined whether fastest racket swing speed was associated with optimal-tension outcomes. Importantly, tied maximum values were handled using a neutral coding rule rather than being systematically assigned to the highest string tension. This approach reduces the risk of upward bias in individualized optimal-tension variables and makes the correlation analyses more statistically defensible.

For both forehand and backhand strokes, fastest racket swing speed was significantly and positively associated with the ball speed obtained at the speed-optimal tension. This indicates that players with greater side-specific speed-production capacity tended to achieve higher ball speeds when each player’s individual fastest-tension condition was considered. However, fastest racket swing speed was not significantly associated with speed-optimal tension itself, accuracy-optimal tension, or the best accuracy score. Thus, a player who swings faster does not necessarily require a higher or lower string tension, nor does faster swing capacity necessarily translate into better landing accuracy.

These findings suggest that maximal swing capacity mainly reflects speed-output potential, whereas optimal string-tension selection and landing control also depend on impact timing, racket-face orientation, contact stability, perceptual feedback, and individual technical characteristics. Sex-controlled sensitivity analyses further indicated that the relationship between swing speed and ball speed was partly influenced by sex-related differences in physical capacity and hitting output. Fastest racket swing speed can therefore be used as a descriptive indicator of tennis-specific speed capacity, but it should not be treated as an independent or sufficient basis for selecting racket string tension (Elliott, 2006; Reid et al., 2013; Fitts, 1954; Schmidt et al., 1979).

### 4.5 Sport-psychological value of perceived comfort and equipment fit

This study evaluated perceived comfort as a subjective equipment-user outcome by using an immediate 5-point comfort rating and a final most-comfortable racket choice. The perceived-comfort measure specified the exact question and response anchors, which supported reproducibility of the subjective assessment. Comfort was assessed after the ball-speed trials because these trials required maximal effort and were expected to generate pronounced ball-racket impact forces and tactile feedback; this timing also helped reduce the possibility that comfort ratings would be influenced by success or failure in the subsequent accuracy task.

The subjective-objective fit analyses showed that the tension perceived as most comfortable was not necessarily the same as the objectively speed-optimal or accuracy-optimal tension. Exact agreement between subjectively preferred tension and speed-optimal tension was limited, especially for the backhand, and agreement with accuracy-optimal tension was also incomplete. In addition, Spearman and sex-controlled partial Spearman analyses showed no FDR-corrected significant associations between subjectively preferred tension and objectively optimal-tension outcomes.

From a sport-psychological perspective, this mismatch is important. Perceived comfort, impact feel, and trust in equipment may influence confidence in the racket and willingness to use a given configuration consistently, but these perceptual judgments do not necessarily identify the objectively optimal tension for speed or accuracy. Perceived comfort should therefore be viewed as a complementary dimension of racket fitting rather than as a substitute for performance testing. At the same time, the psychological interpretation should remain proportionate because perceived comfort was assessed using a single-item rating and final preferred-racket choice rather than a validated multidimensional psychological scale. Future studies could assess perceived control, racket trust, confidence in stroke execution, affective comfort, and intention to continue using a given racket configuration (Bower and Cross, 2003; Stroede et al., 1999; Yeh et al., 2019; Cross, 2015; Ripberger et al., 2025; Piquer-Piquer et al., 2025).

### 4.6 Practical implications and limitations

The present results suggest that racket string-tension selection should be based on a trade-off among ball speed, hitting accuracy, and perceived comfort. For training tasks that emphasize speed production, intermediate tensions may be worth testing, although this should be interpreted cautiously because the overall tension effect for ball speed was not significant. For tasks that emphasize control and landing stability, medium-to-high tensions may be considered because accuracy improved more consistently with increasing tension. Coaches and stringers should combine objective speed and accuracy testing with players’ perceived comfort and individual technical characteristics rather than relying solely on either performance data or subjective feel.

Several methodological limitations should be considered. First, all participants used a two-handed backhand; because single-handed backhands have distinct biomechanical characteristics, the findings may not fully generalize to players using a single-handed backhand. Second, the indoor venue constrained the hitting distance, which was shorter than the standard baseline-to-net distance. Third, although the target board was positioned to keep the effective scoring area above net height, the task still represented a simplified target-hitting environment rather than a full-court rally situation.

Fourth, the ball feeder was configured as a controlled vertical release device without machine-generated horizontal exit velocity or spin. This setup improved standardization and reduced variability in feed speed, direction, and spin, but it did not reproduce the full kinematic complexity of real incoming tennis balls. Fifth, the sample size was relatively small and the sex distribution was unbalanced, so sex-related accuracy patterns should be interpreted as preliminary and exploratory. Sixth, perceived comfort was measured using a single-item scale and final preference choice, which is practical but does not capture the full psychological structure of equipment feel. Future research should include larger and more balanced samples, incorporate single-handed backhand players, evaluate performance over realistic full-court dimensions and more representative incoming-ball conditions, retain trial-level observations for mixed-effects modeling, and use more comprehensive measures of equipment-related perception and confidence (Baayen et al., 2008).

## 5 Conclusion

In trained tennis players, forehand ball speed was higher than backhand ball speed, but the pattern of ball-speed change across string tensions did not differ significantly between forehand and backhand strokes. String tension had a significant effect on hitting accuracy, with higher tensions generally associated with better accuracy, and this effect was moderated by sex. Fastest racket swing speed was significantly associated with the ball speed obtained at the individualized speed-optimal tension, but not with the numerical values of optimal tension or the best accuracy score. Sex-controlled analyses suggested that the relationship between fastest racket swing speed and optimal ball-speed output was partly influenced by sex-related differences. Subjectively preferred tension showed limited agreement with objectively optimal tension conditions and was not significantly associated with objectively speed- or accuracy-optimal outcomes after FDR correction. These findings suggest that perceived comfort is an important sport-psychological and equipment-user outcome, but it should be used in combination with objective performance testing. String-tension selection should balance speed, accuracy, sex-related adaptation, side-specific swing capacity, and perceived comfort rather than relying on a single fixed-tension recommendation or subjective preference alone.

## Data Availability

The raw data supporting the conclusions of this article will be made available by the authors, without undue reservation.
